# Combining inhibitor tolerance and D-xylose fermentation in industrial *Saccharomyces cerevisiae* for efficient lignocellulose-based bioethanol production

**DOI:** 10.1186/1754-6834-6-120

**Published:** 2013-08-26

**Authors:** Mekonnen M Demeke, Françoise Dumortier, Yingying Li, Tom Broeckx, María R Foulquié-Moreno, Johan M Thevelein

**Affiliations:** 1 Laboratory of Molecular Cell Biology, Institute of Botany and Microbiology, KU Leuven, Leuven-Heverlee, Flanders B-3001, Belgium; 2 Department of Molecular Microbiology, VIB, Kasteelpark Arenberg 31, Leuven-Heverlee, Flanders B-3001, Belgium

**Keywords:** Bioethanol production, Pentose utilization, Inhibitor tolerance, *Saccharomyces cerevisiae*, Meiotic recombination, Spruce hydrolysate, Very-high gravity fermentation

## Abstract

**Background:**

In addition to efficient pentose utilization, high inhibitor tolerance is a key trait required in any organism used for economically viable industrial bioethanol production with lignocellulose biomass. Although recent work has succeeded in establishing efficient xylose fermentation in robust industrial *Saccharomyces cerevisiae* strains, the resulting strains still lacked sufficient inhibitor tolerance for efficient sugar fermentation in lignocellulose hydrolysates. The aim of the present work was to combine high xylose fermentation activity and high inhibitor tolerance in a single industrial yeast strain.

**Results:**

We have screened 580 yeast strains for high inhibitor tolerance using undetoxified acid-pretreated spruce hydrolysate and identified a triploid industrial baker’s yeast strain as having the highest inhibitor tolerance. From this strain, a mating competent diploid segregant with even higher inhibitor tolerance was obtained. It was crossed with the recently developed D-xylose fermenting diploid industrial strain GS1.11-26, with the Ethanol Red genetic background. Screening of 819 diploid segregants from the tetraploid hybrid resulted in two strains, GSF335 and GSF767, combining high inhibitor tolerance and efficient xylose fermentation. In a parallel approach, meiotic recombination of GS1.11-26 with a haploid segregant of Ethanol Red and screening of 104 segregants resulted in a similar inhibitor tolerant diploid strain, GSE16. The three superior strains exhibited significantly improved tolerance to inhibitors in spruce hydrolysate, higher glucose consumption rates, higher aerobic growth rates and higher maximal ethanol accumulation capacity in very-high gravity fermentation, compared to GS1.11-26. In complex medium, the D-xylose utilization rate by the three superior strains ranged from 0.36 to 0.67 g/g DW/h, which was lower than that of GS1.11-26 (1.10 g/g DW/h). On the other hand, in batch fermentation of undetoxified acid-pretreated spruce hydrolysate, the three superior strains showed comparable D-xylose utilization rates as GS1.11-26, probably because of their higher inhibitor tolerance. They produced up to 23% more ethanol compared to Ethanol Red.

**Conclusions:**

We have successfully constructed three superior industrial *S. cerevisiae* strains that combine efficient D-xylose utilization with high inhibitor tolerance. Since the background strain Ethanol Red has a proven record of successful industrial application, the three new superior strains have strong potential for direct application in industrial bioethanol production.

## Background

Biofuels produced from non-food lignocellulosic biomass, such as agricultural and forest residues, municipal solid wastes and energy crops are believed to be an important sustainable solution for the future transport energy deficit and the green house gas emission problem [1]. Such lignocellulosic materials constitute the most abundant organic materials in the biosphere and thus represent a huge and renewable reservoir for transport energy [2]. Although important technological advances have been realized to exploit this potential in the last three decades, so-called second-generation biofuel production is not yet feasible in an economically viable way [3,4].

Bio-ethanol is currently the dominant renewable biofuel used in the transportation sector [5]. It has already been introduced on a large scale in various countries, such as Brazil, the US, and increasingly in European countries, and is now predominantly produced from food crops. In the past few years, substantial efforts have been focused on production of bioethanol from non-food lignocellulose biomass. Bioethanol production from lignocellulosic wastes, such as crop residues and sugar cane bagasse, and from cultivation of bioenergy crops, has the potential to contribute significantly to the replacement of fossil fuel for transportation purposes [6,7].

The main challenges in advanced bioethanol production are the development of efficient and cheap technologies to liberate all fermentable sugars from lignocellulosic feedstocks, and the engineering of robust microorganisms able to rapidly ferment all sugar present in the biomass hydrolysate, mainly glucose and D-xylose [8,9]. Several pretreatment and enzymatic hydrolysis processes have been reported with increasing efficiency for releasing the sugars from the biomass [10-12]. Yeast strains have even been developed that secrete cellulolytic enzymes for use in consolidated bioprocessing [13]. However, in addition to the release of fermentable sugars, large amounts of several types of inhibitory compounds are released during the pretreatment process. They inhibit microbial fermentation and growth, resulting in severely reduced ethanol yield and productivity. Therefore, economically viable industrial production of lignocellulose-derived bioethanol requires not only a microorganism that is able to ferment all hexose and pentose monosaccharides in the lignocellulose hydrolysates, but also exhibits unusually high tolerance to the toxic compounds present in the lignocellulose hydrolysates.

Substantial progress has been made in the past few years to develop yeast strains that are able to ferment D-xylose [14-19], and to obtain strains with improved inhibitor tolerance [20-22]. Some D-xylose fermenting recombinant strains of *S. cerevisiae* and natural D-xylose utilizing yeast species with improved inhibitor tolerance have also been reported [23,24]. However, most of this work has been performed with laboratory *S. cerevisiae* strains or strains of *S. cerevisiae* and other yeast species without proven track record in industrial bioethanol production. In addition, the performance of the best strains available in terms of D-xylose fermentation and inhibitor tolerance still requires much improvement in order to reach efficient fermentation of lignocellulosic hydrolysates, especially at a higher solid loading [25]. Since pentose fermentation appears to be much more sensitive to the toxic inhibitors [26], the productivity of the yeast in high-density lignocellulose hydrolysates is largely determined by the robustness of the pentose fermentation.

Recently, a D-xylose fermenting strain GS1.11-26 has been developed from Ethanol Red, a prime industrial yeast strain used in first-generation bioethanol production with corn and wheat [27]. Ethanol Red has a proven track record of excellent fermentation capacity and yield, high robustness and stress tolerance, excellent performance in fed-batch production on molasses, tolerance to dehydration and maintenance of high vitality during storage and transport. For that reason, the strain GS1.11-26 was considered to have very promising potential for development of an all-round robust yeast strain for efficient fermentation of various lignocellulosic materials. However, due to the accumulation of background mutations during the mutagenesis and/or evolutionary engineering procedures used to develop the strain, GS1.11-26 did not retain the same tolerance to high concentrations of ethanol and acetate, and showed reduced ethanol accumulation capacity in very high-density fermentations compared to the original Ethanol Red strain. Moreover, it also had a partial respiratory defect causing a reduced aerobic growth rate, which would compromise large-scale propagation of yeast in fed-batch mode [27]. Hence, as such the strain would not be suitable for direct industrial application.

We now report the development of three new xylose-utilizing industrial yeast strains, derived from the GS1.11-26 strain, and which lack its negative properties. The new strains are diploid and were obtained through meiotic recombination with a diploid segregant from a strongly inhibitor-tolerant triploid strain and with a haploid segregant of Ethanol Red. The first strain was the most inhibitor tolerant strain identified by screening a large collection of yeast strains for tolerance to undetoxified acid-pretreated spruce hydrolysate. The three new superior strains exhibited significantly increased tolerance to various inhibitors in spruce hydrolysate, faster growth rate in glucose medium and a faster glucose consumption rate and higher ethanol accumulation capacity in very high gravity fermentations. The maximum D-xylose utilization rate of the three new strains was slower than that of GS1.11-26, but they completely consumed 37 g/L D-xylose and 36 g/L glucose in about 32 h. Our results also demonstrate that commercially important traits present in diploid industrial yeast strains can be combined into a single industrial yeast strain with superior properties and performance without the need for isolation of haploid derivatives.

## Results

### Screening of *S. cerevisiae* strain collection for tolerance to inhibitors in spruce hydrolysate

We first aimed at obtaining a strain with extremely high performance in terms of growth and fermentation directly in inhibitor-rich lignocellulose hydrolysate, since simultaneous tolerance to multiple inhibitors is important for high productivity in lignocellulose hydrolysates [28]. For that purpose, undetoxified acid-pretreated spruce hydrolysate was used as medium for screening, because this hydrolysate contains more inhibitors and in higher concentrations than most other hydrolysates [29,30]. Using this medium, we screened 580 different *S. cerevisiae* strains first for the ability to grow in different concentrations of spruce hydrolysate. The strain collection consisted of laboratory strains, industrial strains (wine, beer, baker’s and bioethanol production strains) as well as a variety of natural isolates from various sources. The first screening was done with growth tests on YP-agar plates containing up to 70% whole slurry of the spruce hydrolysate at pH 5.5.

**Figure 1:**
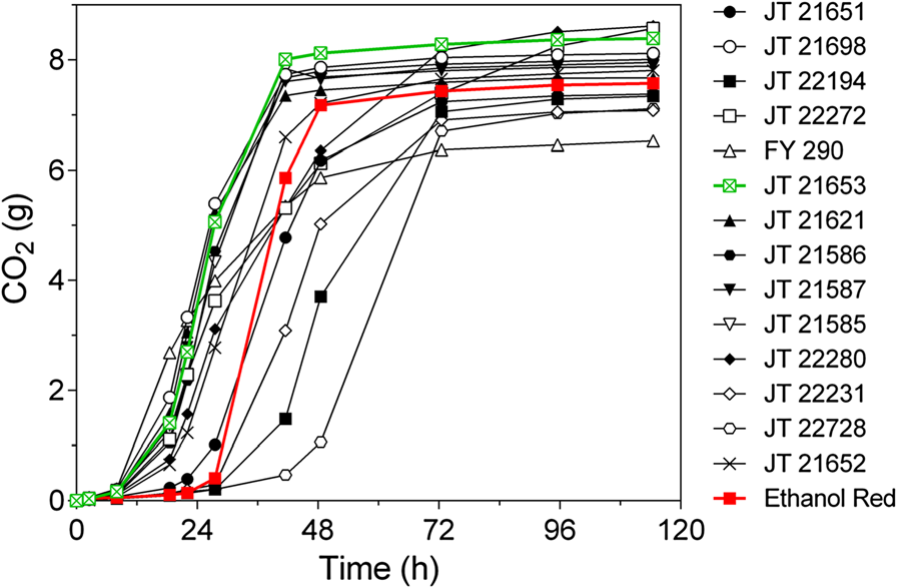
**Fermentation performance of the 14 different preselected inhibitor tolerant yeast strains in comparison to Ethanol Red.** Semi-anaerobic fermentation was performed in 30% pretreated slurry of spruce hydrolysate supplemented with YP and glucose to 200 g/L at pH 5.5. The course of the fermentation was followed by weight loss due to CO_2_ production during the fermentation. The robust industrial strain Ethanol Red (red) was used as a reference, strain JT21653 (green) was selected as the best performing strain.

**Figure 2:**
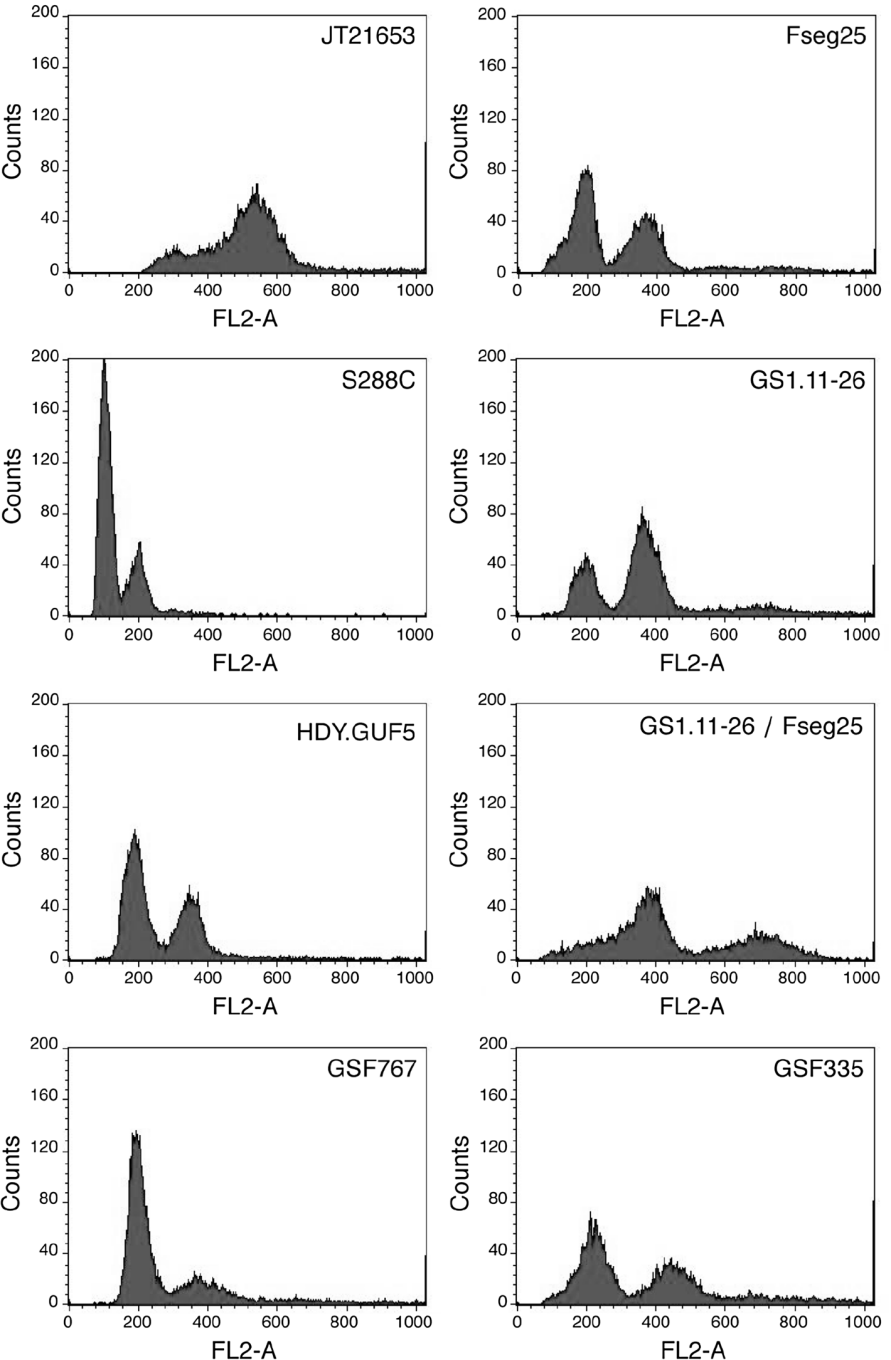
**Determination of DNA content by flow cytometry.** Strains were grown to exponential phase, after which they were fixed with ethanol, and the DNA was stained with propidium iodide. Control haploid (S288c) and diploid (HDY.GUF5) strains were used for comparison.

From the 580 strains tested, 35 strains that performed the best in the plate test were subsequently screened in more detail by determining their fermentation performance in 30% spruce hydrolysate supplemented with YP and glucose to 200 g/L, at pH 5.5. The high concentration of glucose was used to obtain high osmotic stress at the beginning and high ethanol stress at the end of the fermentation. The industrial strain Ethanol Red was used as control. Out of the 35 strains tested, we selected 14 strains that showed at least similar fermentation performance as the industrial strain Ethanol Red. Finally, the fermentation performance of the 14 best strains was tested again in a single fermentation experiment and ten strains were found to perform consistently better than Ethanol Red. They mainly showed a shorter lag phase (Figure 1). The best strain, JT21653, which showed both a shorter lag phase and a more complete attenuation of the sugar, was chosen for further analysis. JT21653 is a baker’s yeast that was purchased from a local commercial source. A species identification test was performed on a single cell isolate at the BCCM/MUCL (Mycothèque de l'Université Catholique de Louvain, Louvain-la-Neuve, Belgium), which confirmed that the yeast was *S. cerevisiae* Meyen ex. E.C. Hansen*.* Flow cytometry analysis of the DNA content revealed that JT21653 had a triploid genome (Figure 2). The strain showed good sporulation and high spore viability and hence, was selected for further analysis.

### Isolation of an inhibitor tolerant segregant of JT21653

**Figure 3:**
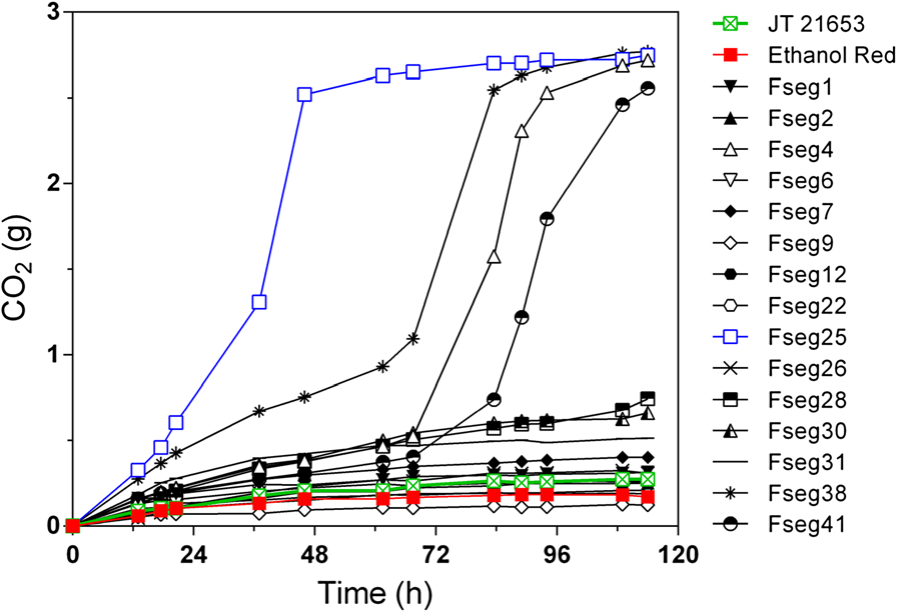
**Selection of a superior segregant from the inhibitor tolerant baker’s yeast JT21653.** Fermentation was performed in semi-anaerobic conditions using treated slurry of spruce material at 77% concentration and supplemented with 70 g/L glucose. CO_2_ production was estimated from the weight loss. Fseg25 (blue) showed the best performance, with in particular a very short lag phase compared to the other segregants. The diploid parent JT21653 (green) and the industrial bioethanol production strain Ethanol Red (red) as well as all other segregants, except for four, were not able to ferment appreciably at this concentration of spruce hydrolysate within 120 h.

In order to select a segregant of JT21653 with an inhibitor tolerance at least as good as the triploid parent, we screened 41 segregants of JT21653 for fermentation performance under semi-anaerobic conditions in 40% spruce hydrolysate supplemented with YP and glucose to 200 g/L at pH 5.5 (results not shown). From this prescreening, we selected the 15 segregants with the best fermentation performance. For selection of the most inhibitor-tolerant segregant, the concentration of the spruce hydrolysate was increased to 77% and supplemented with 70 g/L glucose. This concentration of spruce hydrolysate was severely inhibitory to the industrial strain Ethanol Red, the triploid parent JT21653 as well as the majority of the 15 segregants, whereas four segregants were able to complete the fermentation under these conditions albeit with varying rates (Figure 3). One segregant, named Fseg25, performed exceptionally well, initiating the fermentation with virtually no lag phase and completing it already after about 48 h, a time at which only other segregant had made a significant start-up of the fermentation. Analysis of the DNA content of Fseg25 by flow cytometry indicated that the strain had a diploid genome (Figure 2). In addition, it was able to mate with a *MATα/α* strain, indicating that it was *MAT****a/a*** and the resulting tetraploid strain produced viable spores. Therefore, the Fseg25 segregant was chosen as a mating partner for genetic recombination with the diploid *MATα/α* strain GS1.11-26.

### Meiotic recombination of GS1.11-26 with Fseg25

To combine the superior D-xylose fermentation performance of GS1.11-26 with the superior inhibitor tolerance trait of Fseg25, we mated the *MATα/α* strain GS1.11-26 with the *MAT****a/a*** segregant Fseg25. The tetraploid strain generated was sporulated, and 819 meiotic segregants were screened in order to select diploid hybrid F1 segregants with the required properties, mainly efficient D-xylose fermentation, fast aerobic growth and high inhibitor tolerance in spruce hydrolysate.

### Screening for D-xylose fermentation capacity

We first performed a prescreening of the 819 segregants based on their ability to grow on YPX solid medium, in order to reduce the number of strains to be evaluated in fermentations. All segregants that showed detectable growth on solid xylose medium were further analyzed for growth in liquid medium. For that purpose, the strains were inoculated in 1 mL YPX medium at an initial OD_600_ of 1.0. After about 24 h incubation, a range of cell densities, from OD_600_ of about 5 up to 33 was observed for the different segregants. Strain GS1.11-26 showed an OD_600_ between 28 and 33 in different replicate growth assays. To monitor the correlation between growth in liquid YPX and fermentation performance, segregants growing to an OD_600_ above 5 were evaluated by fermentation in YP medium containing 40 g/L D-xylose. We observed that, most of the best D-xylose fermenting strains also performed well in such growth evaluation experiments (data not shown). Thus, the majority of poor D-xylose fermenting segregants could be excluded by using a cut-off value for growth to an OD_600_ of 15 in 24 h, since all the good D-xylose fermenting segregants grew to an OD_600_ of above 15. Hence, growth in liquid YPX medium for 24 h and selection of the segregants growing to a minimum OD_600_ of 15 was considered to be the best method for rapid initial screening and elimination of poor performers.

**Figure 4:**
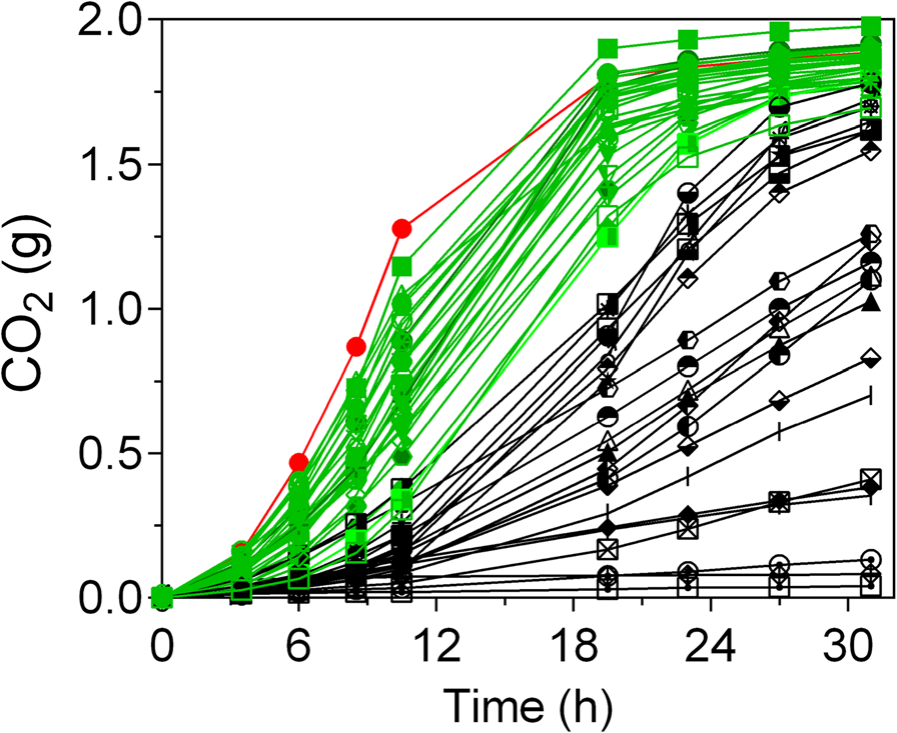
**D-xylose fermentation profile of the 48 preselected D-xylose utilizing segregants obtained from the tetraploid strain GS1.11-26/Fseg25, in YP medium containing 40 g/L D-xylose.** The 27 selected segregants with the best performance are shown in green and the parent strain GS1.11-26 is shown in red. A similar fermentation profile was obtained during the preselection step in the same conditions.

Using this method, about 168 segregants growing to OD_600_ values of about 15 in 24 h were preselected and further tested for D-xylose fermentation performance in semi-anaerobic conditions. This was done in different batches of experiments (results not shown) and finally resulted in 48 segregants with moderate to rapid D-xylose fermentation capacity (Figure 4). To allow a proper comparison, the 48 selected segregants were evaluated in a single batch of fermentation experiments. The 27 best segregants, with a D-xylose fermentation performance close to that of GS1.11-26, were eventually selected for further analysis (Figure 4). Flow cytometry analysis showed that the selected 27 segregants all had a DNA content similar to that of a diploid control strain (data not shown). Hence, all segregants appeared to be diploid strains, although aneuploidy for one or more chromosomes cannot be ruled out.

### Screening of hybrid segregants for aerobic growth rate and inhibitor tolerance

**Figure 5:**
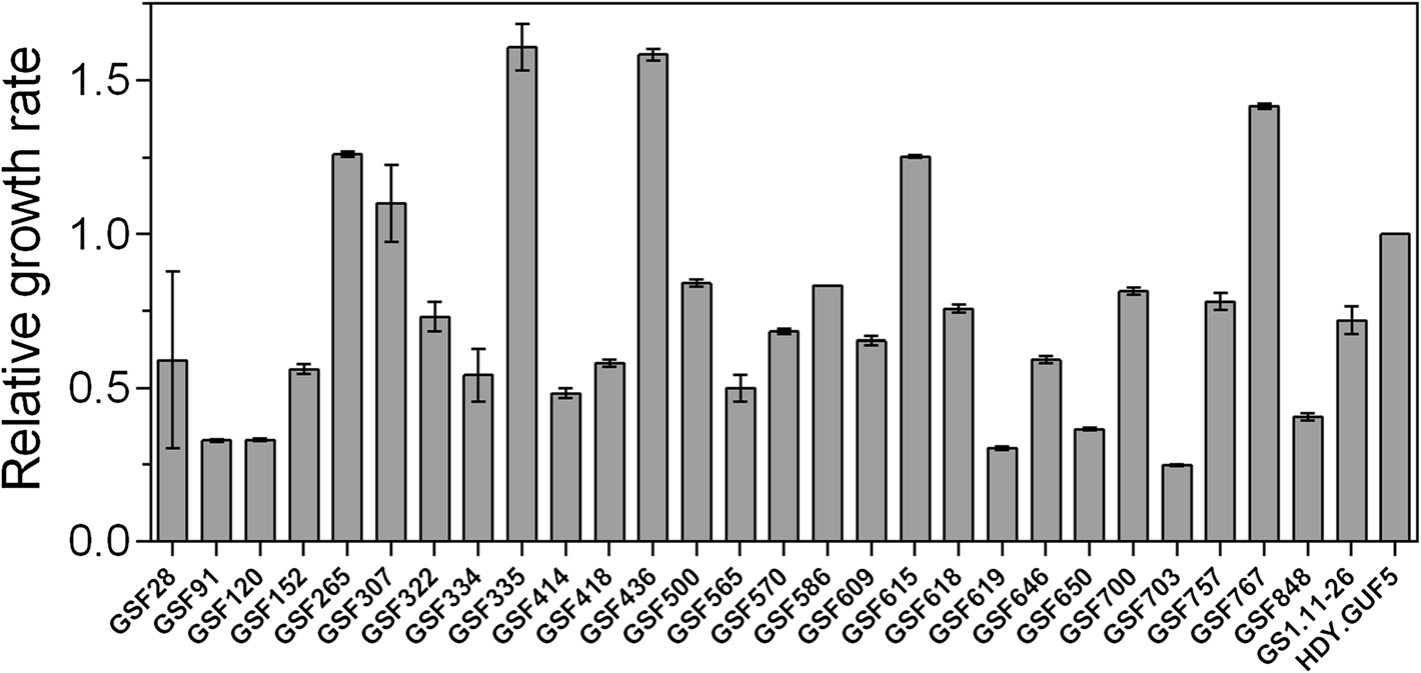
**Relative maximum growth rate of the 27 best D-xylose fermenting segregants in comparison to the strain HDY.GUF5.** Growth was performed in synthetic medium containing 20 g/L glucose in 200 μl volume using a bioscreen assay at an initial OD_600_ of 0.1. Error bars represent standard deviation from average values of triplicate experiments. The maximum growth rate obtained for each strain was calculated relative to HDY.GUF5.

One of the shortcomings of GS1.11-26 for direct industrial application was the slow aerobic growth rate, which is a key factor for yeast propagation in the preparation of sufficient inoculum to start the industrial fermentation process. Hence, we screened the 27 D-xylose fermenting strains for growth rate in synthetic medium containing glucose as a carbon source. The recombinant strain HDY.GUF5 (Ethanol Red background) was used as a reference throughout this work since GS1.11-26 was developed from this strain background [27]. From the 27 segregants, only six showed a growth rate as high as that of the HDY.GUF5 strain (Figure 5). When these six segregants were evaluated in inhibitor-rich spruce hydrolysate, only two segregants, named GSF335 and GSF767, grew better than the HDY.GUF5 strain (data not shown). Both strains were found to be diploid and had *MATα/α* mating type. They were selected for further evaluation.

### Backcrossing the diploid GS1.11-26 with a haploid segregant of Ethanol Red

In a parallel approach, the *MATα/α* diploid strain, GS1.11-26, with high D-xylose fermentation activity, was crossed with the segregant ER17 of Ethanol Red. ER17 was previously selected among a number of segregants for its higher acetic acid tolerance in fermentation, which was even better than that of its diploid parent strain Ethanol Red (Meijnen et al., unpublished data).

The crossing of GS1.11-26 with ER17 resulted in a triploid strain. Meiotic segregation in triploid strains often produces two haploid and two diploid progeny in a tetrad [31]. Obtaining diploid progeny is important since haploid strains are unattractive for industrial application, because of their lower genetic stability and robustness [32]. The triploid strain was sporulated and 104 segregants were isolated. Most of the tetrads produced four viable spores. All the isolates were then evaluated for the important phenotypic traits.

### Screening of the hybrid segregants from GS1.11-26/ER17 for growth in D-xylose medium

**Figure 6:**
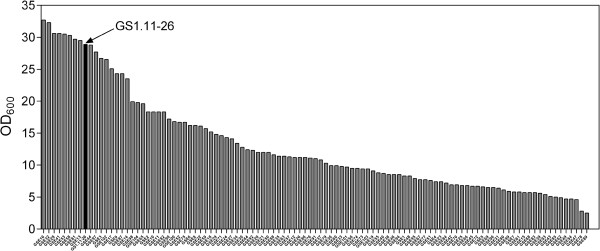
**Screening of 104 segregants obtained from the triploid strain GS1.11-26/ER17, for growth in 1 mL YP medium containing 20 g/L D-xylose as carbon source.** Cells were inoculated at an OD_600_ value of 1.0. The final OD_600_ was then measured after 20 h. The parent strain GS1.11-26 was used as a reference.

The 104 segregants isolated from GS1.11-26/ER17 were first screened for growth in YPX medium as a preselection step. A range of final OD_600_ values from about 2 to 33 was observed (Figure 6), indicating the involvement of multiple genetic factors for D-xylose growth. Since growth on and fermentation of D-xylose have previously shown a good correlation, the 21 segregants that grew best in D-xylose medium were selected for the next evaluation.

### Screening of the hybrid segregants from GS1.11-26/ER17 for aerobic growth rate

The 21 best D-xylose growing segregants were further tested for growth rate in glucose medium. They were pregrown in 3 mL YPD medium. Segregants that yielded after 16 h much lower biomass than HDY.GUF5 were excluded. The remaining seven segregants were tested for growth rate in synthetic medium containing 20 g/L glucose. Two segregants that produced after 16 h lower OD_600_ values than HDY.GUF5 were included for comparison.

**Figure 7:**
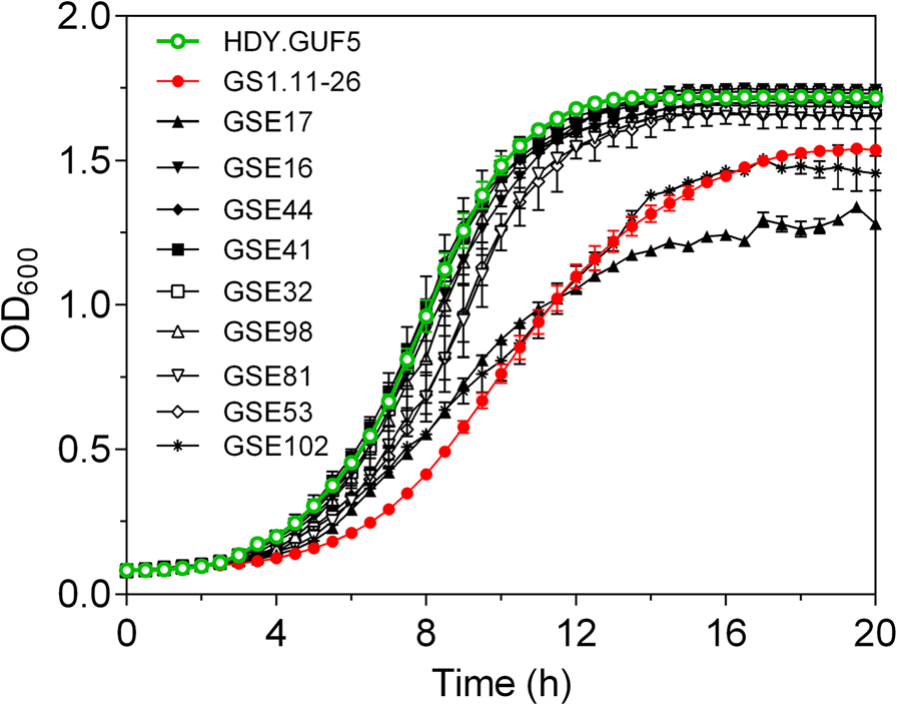
**Screening of the best D-xylose growing segregants, obtained from the triploid strain GS1.11-26/ER17, for aerobic growth rate in glucose medium.** A bioscreen assay was performed in 200 μl volume synthetic medium containing 20 g/L glucose, at an initial OD_600_ of 0.1. Error bars represent standard deviation from the average in triplicate experiments.

In the growth rate assay, seven of the nine strains tested showed a much faster growth rate than GS1.11-26 (Figure 7). The two segregants, GSE17 and GSE102, that produced lower OD_600_ values during the preselection step, also showed a slower growth rate than GS1.11-26, confirming the results from the pregrowth assay. The seven segregants that grew with a rate close to that of HDY.GUF5 were then selected for the final fermentation experiment in D-xylose medium.

### Selection of the most superior D-xylose fermenting and inhibitor tolerant hybrid strains

**Figure 8:**
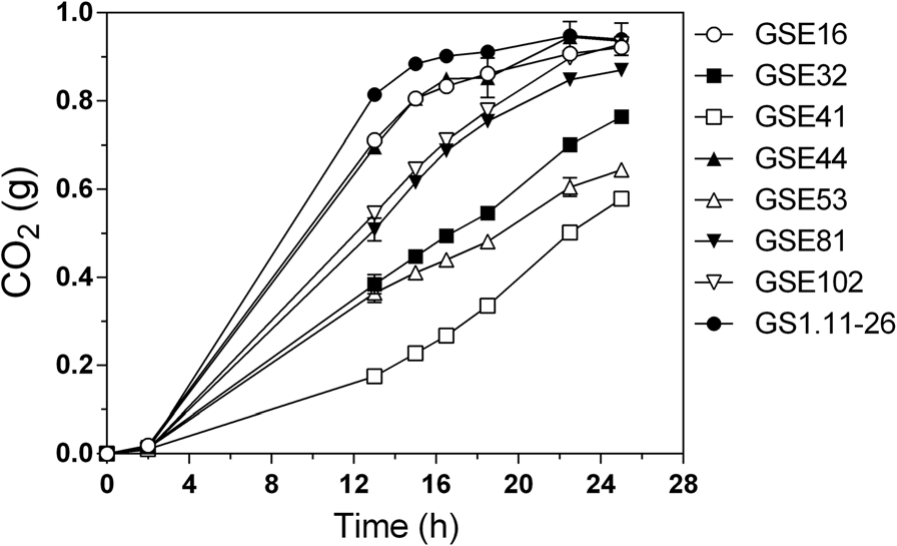
**Selection of efficient D-xylose fermenting segregants from the seven segregants with best performance, obtained from the triploid strain GS1.11-26/ER17.** The fermentations were performed in 50 mL volume with YP medium containing 40 g/L D-xylose. The CO_2_ release was estimated from the weight loss during fermentation. Error bars represent standard deviation from the average in duplicate experiments.

To select first the best D-xylose fermenting strains, the seven hybrid strains with a rapid growth rate in glucose were evaluated for fermentation performance in 50 mL YP medium containing 40 g/L D-xylose as a sole carbon source. The original evolved strain GS1.11-26 was used for comparison. As can be seen in Figure 8, two strains, GSE44 and GSE16, utilized D-xylose very well, nearly as well as GS1.11-26. Their rate of D-xylose utilization (estimated from the CO_2_ production rate) was slightly slower than that of GS1.11-26.

These two strains, GSE44 and GSE16, were tested for tolerance to acetic acid and to spruce hydrolysate. GSE16 showed similar tolerance towards acetic acid and inhibitors in spruce hydrolysate as the HDY.GUF5 strain (growth in liquid YPD containing 6 g/L acetic acid, pH4.5, or 80% of the liquid portion of spruce hydrolysate, pH 5). On the other hand, GSE44 displayed reduced tolerance especially to acetic acid, showing growth in the presence of only up to 4 g/L acetic acid and no growth anymore at 5 g/L acetic acid (results not shown). Hence, the diploid strain GSE16, which is *MAT****a****/α*, was selected for further evaluation.

### Evaluation of the most superior hybrid strains

#### Fermentation performance with a glucose-D-xylose mixture

**Figure 9:**
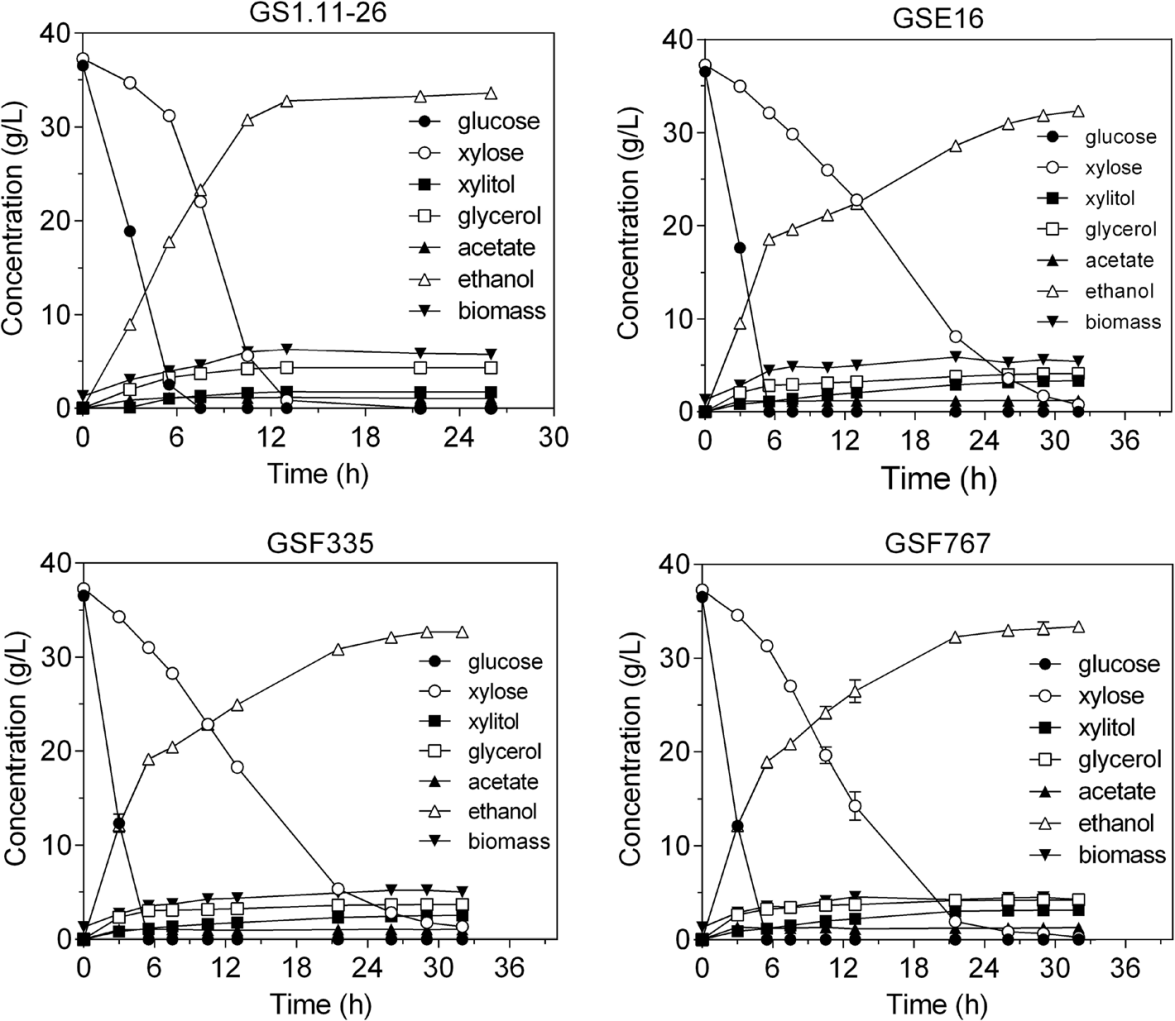
**Fermentation performance of the three new hybrid strains in glucose and D-xylose mixture in comparison to that of GS1.11-26.** The fermentations were performed in YP medium containing 37 g/L glucose and 37 g/L D-xylose, with an initial biomass of 1.3 g/L. Error bars represent standard deviation from the average in duplicate experiments.

The three selected strains, GSE16 (obtained from backcrossing GS1.11-26 with a segregant of Ethanol Red), GSF335 and GSF767 (obtained from crossing GS1.11-26 with a segregant of JT21653), were evaluated in a more controlled fermentation experiment in 100 mL YP medium containing 36 g/L glucose and 37 g/L D-xylose at 35°C. The inoculum cell density was 1.3 g DW/L. The performance of the three hybrid strains was compared with that previously reported for the strain GS1.11-26. GS1.11-26 completely consumed both D-xylose and glucose in about 13 h, compared to about 32 h for the other three strains [27] (Figure 9).

**Figure 10:**
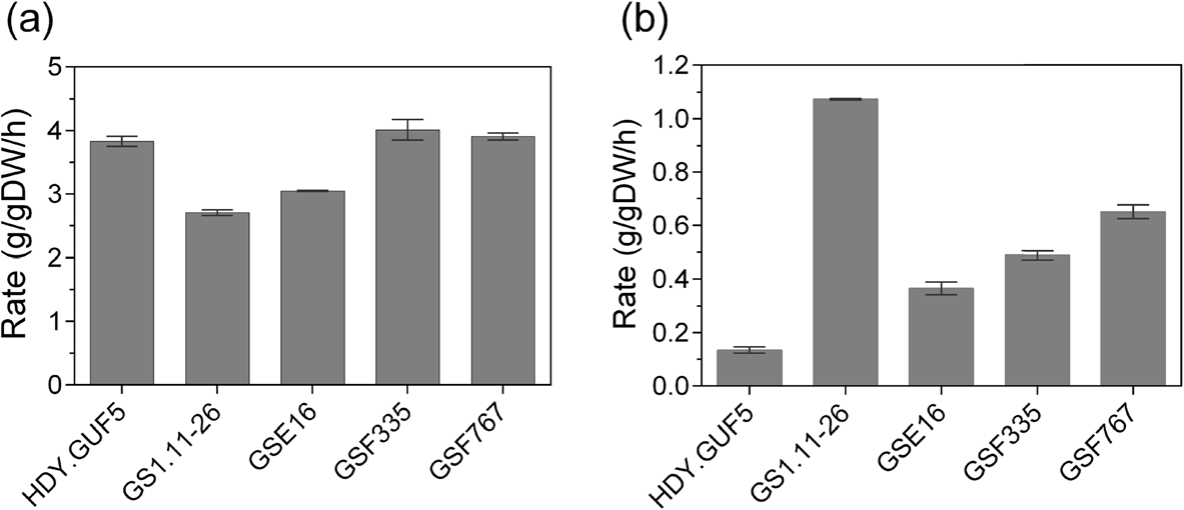
**Maximum sugar consumption rates attained by the three new hybrid strains and GS1.11-26. (a)**, maximum glucose consumption rate; **(b)**, maximum D-xylose consumption rate. The values were calculated from the fermentation experiment shown in Figure 9. Maximum D-xylose utilization rate was obtained after glucose exhaustion. Error bars represent standard deviation from the average in duplicate experiments.

The glucose consumption rate with the three new hybrid strains was higher than that of the evolved strain GS1.11-26 (Figures 9 and 10a). Moreover, GSF335 showed even a slightly higher glucose consumption rate than the original parent strain HDY.GUF5 (Figure 10a). However, GS1.11-26 remained superior in terms of D-xylose consumption rate and ethanol productivity from D-xylose (Figures 9 and 10b). Among the three new hybrid strains, GSF767 showed the highest D-xylose consumption rate (0.65 g/g DW/h), but its maximum D-xylose utilization rate was still about 40% lower than that of GS1.11-26 (1.10 g/g DW/h). On the other hand, the same ethanol yield was obtained with GS1.11-26 and GSF767 (0.46 g/g initial sugar or 90.2% of the theoretical maximum), while it was slightly lower with the other two strains GSF335 and GSE16 (0.44 g/g initial sugar or 86.3% of the theoretical maximum). Partial co-utilization of D-xylose and glucose was observed with all three new hybrid strains as well as with GS1.11-26, as previously reported [27] (Figure 9).

#### Fermentation performance in inhibitor-rich spruce hydrolysate

**Figure 11:**
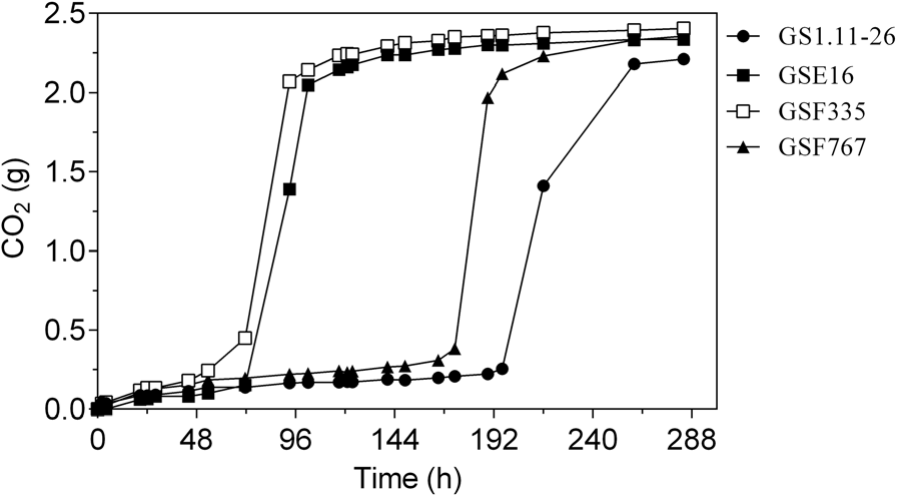
**Inhibitor tolerance assay in acid-pretreated spruce hydrolysate with the three new hybrid strains in a small-scale fermentation experiment.** The fermentation was performed using the whole slurry of spruce hydrolysate (60%) supplemented with 40 g/L glucose with continuous stirring at 200 rpm. Cells were inoculated at an initial cell density of 1.3 g DW/L from a stationary phase preculture in YPD medium. The CO_2_ production was estimated from the weight loss during fermentation.

The three new hybrid strains, GSE16, GSF335 and GSF767, were evaluated for inhibitor tolerance in a fermentation experiment with acid pretreated spruce material. The whole slurry of the spruce material, 60%, supplemented with yeast extract and peptone, was used. Glucose (40 g/L) was added since the sugar concentration in the hydrolysate was rather low (about 13 g/L), which would not allow a proper comparison of the fermentation performance between the strains. Under these conditions, the rate of fermentation (as estimated from CO_2_ release) by GSF335 and GSE16 was much faster than that of GS1.11-26 (Figure 11). GSF767 also performed slightly better than GS1.11-26. This result indicates that the new hybrid strains, especially GSF335 and GSE16, have significantly better tolerance than GS1.11-26 towards the inhibitors present in acid pretreated spruce hydrolysate.

**Figure 12:**
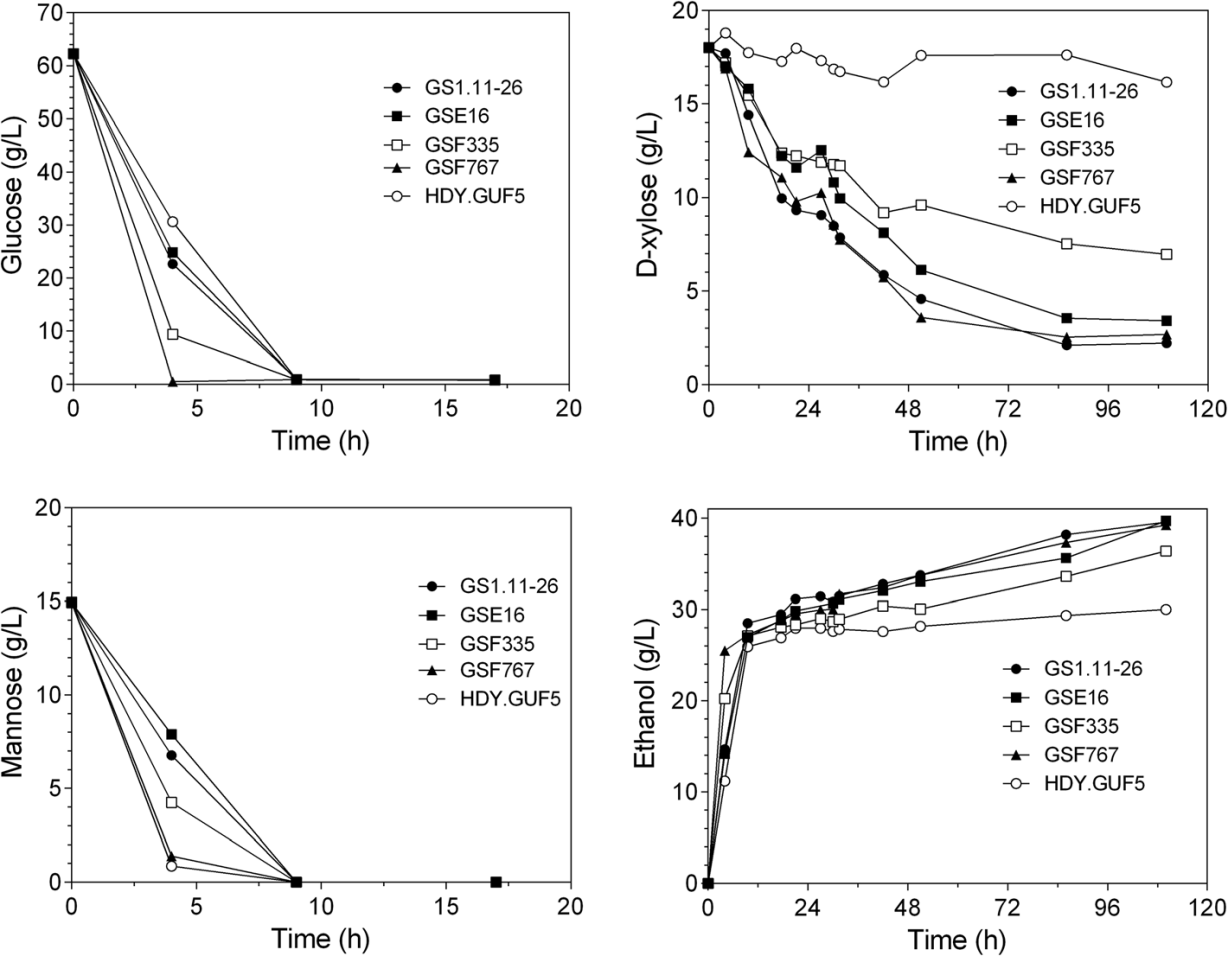
**Fermentation profile of the three new hybrid strains in semi-anaerobic batch fermentation with acid-pretreated spruce hydrolysate.** The fermentation was performed with acid-pretreated spruce hydrolysate at a solid loading of 11%, supplemented with yeast nitrogen base, ammonium sulfate and amino acids. The final glucose and D-xylose concentrations were adjusted to 62 g/L and 18 g/L, respectively. The mannose was only derived from the hydrolysate. Fermentation was started at an initial density of 4 g DW/L and carried out with continuous stirring at 200 rpm.

To evaluate more precisely the D-xylose and glucose utilization rates in the spruce hydrolysate, we studied the performance of the three strains in a similar batch fermentation using undetoxified spruce hydrolysate at a solid loading of 11% (corresponding to about 50% of the slurry), and supplemented with glucose and D-xylose to a final concentration of 62 g/L and 18 g/L, respectively. The fermentation was performed at an initial cell density of 4 g DW/L (Figure 12). Glucose and mannose (derived from the hydrolysate) were completely consumed in less than 10 h by all three strains as well as by the control strains HDY.GUF5 and GS1.11-26, with GSF767 showing the fastest glucose consumption rate, using all glucose in 4 h. The D-xylose fermentation rate was much slower compared to that of glucose fermentation and was now much more similar for all strains than in the more concentrated hydrolysate (except HDY.GUF5, which cannot use D-xylose). The hybrid strain GSF767 showed a similar D-xylose fermentation rate to that of GS1.11-26, whereas the rate was slightly lower for GSE16 and GSF335. No xylitol and little glycerol (about 0.05 g/g) was produced by the three hybrid strains and GS1.11-26 (results not shown). Although the D-xylose utilization rate was slower, the final ethanol concentration and yield obtained by the hybrid strains and GS1.11-26 was comparable. Compared to HDY.GUF5, the two hybrid strains GSE16 and GSF767 as well as GS1.11-26 produced about 23% more ethanol, due to their efficient D-xylose utilization. The ethanol yield of GSF335 was lower, but still 13% higher than that of HDY.GUF5.

#### Fermentation performance in very-high gravity fermentation

**Figure 13:**
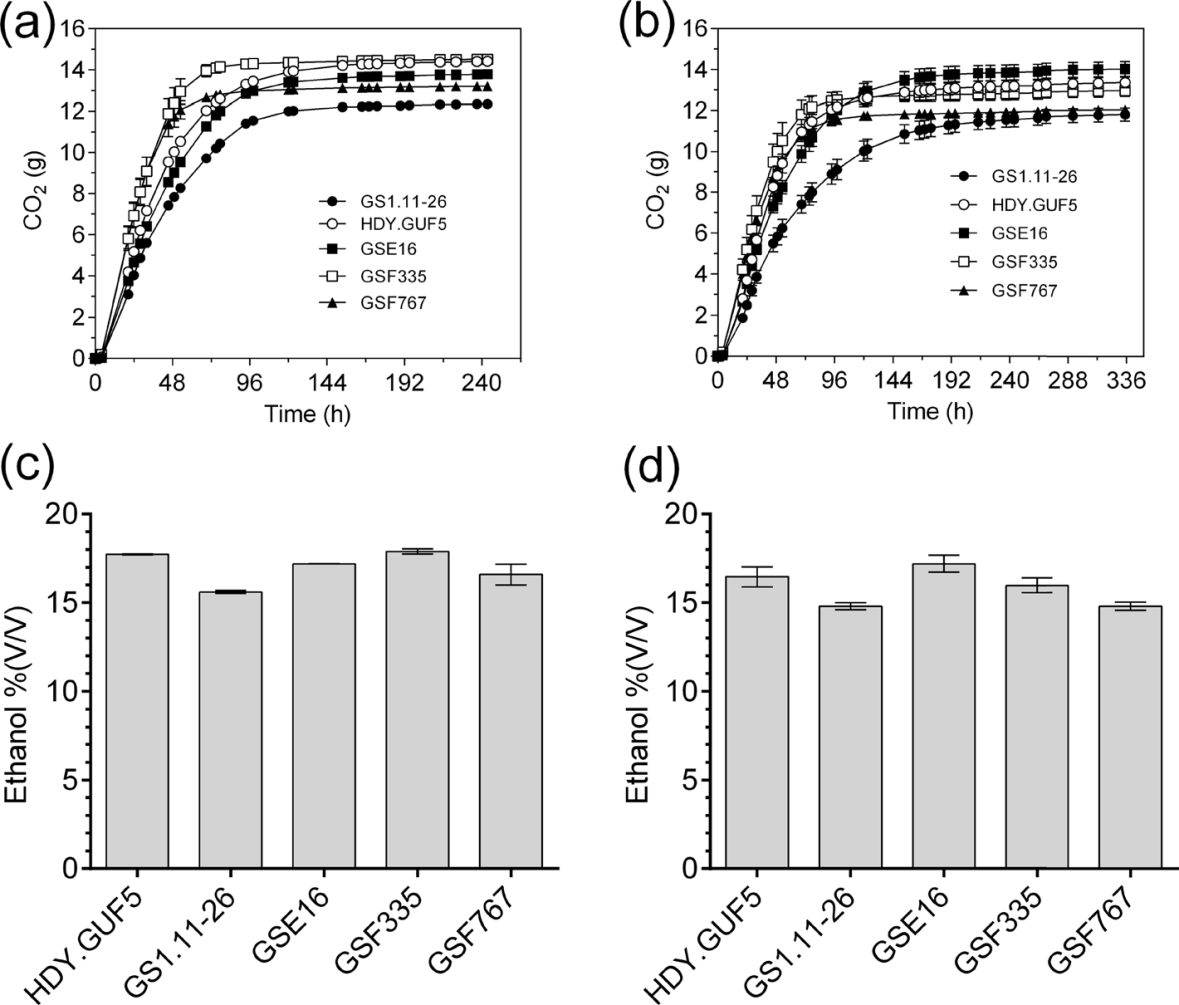
**Very-high gravity fermentation in YP with 330 g/L glucose. (a)** and **(b)** show the fermentation profile in continuous stirring and static conditions, respectively. The figures in **(c)** and **(d)** show the final ethanol titer produced during fermentation of **(a)** and **(b)**, respectively. Fermentation was started at an initial cell density of 1.3 g DW/L.

The hybrid strains, GSE16, GSF335 and GSF767, were also evaluated for fermentation performance in very-high gravity fermentations using YP medium containing 330 g/L glucose. The wild type, HDY.GUF5, and the evolved strain, GS1.11-26, were included as controls. The fermentation was performed under conditions of continuous stirring at 120 rpm and under mainly static conditions, with only 4 h of stirring in the beginning. Under both conditions, the three hybrid strains performed much better than GS1.11-26 (Figure 13).

Under continuous stirring, GSF335 showed the fastest rate of fermentation (Figure 13a). It reached the same final ethanol concentration as HDY.GUF5, but about 96 h earlier. Moreover, GSF335 also showed the highest rate of fermentation under static fermentation conditions. However, the highest final ethanol titer in static conditions was reached by GSE16 (Figure 13b and d). GSF767 performed similar to HDY.GUF5 for fermentation rate, but reached a somewhat lower final ethanol titer under both conditions. Overall, the three hybrid strains performed much better than GS1.11-26 under both static and continuous stirring conditions. GSF335 showed superior performance especially in the stirred fermentation conditions accumulating the highest ethanol concentration in only 72 h. In a separate very-high gravity fermentation experiment, the final ethanol titer accumulated by GSF335 was higher than that of both the tetraploid strain GS1.11-26/Fseg25 and the diploid strain Fseg25 (results not shown).

## Discussion

The goal of the present paper was to develop a superior D-xylose utilizing industrial yeast strain, lacking the negative properties introduced in the background of the previously reported strain, GS1.11-26, during the mutagenesis and/or evolutionary engineering procedures used to obtain the strain. The development of the GS1.11-26 strain has shown that introduction of efficient D-xylose fermentation capacity in an industrial yeast strain requires multiple genetic modifications [27]. Hence, just like stress and inhibitor tolerance, efficient D-xylose fermentation is a polygenic trait and the engineering of such traits for development of superior industrial yeast strains has been a major challenge for rational engineering strategies [33-36]. As a result, most progress in this field has been made up to now with unbiased approaches, like mutagenesis, selection and evolutionary engineering, where the underlying genetic changes responsible for the improvement in performance generally remain unknown [16,17,23,24,37-43].

In the present study, we describe the construction of three robust industrial yeast strains that combine efficient D-xylose fermentation with very high inhibitor tolerance. This was achieved by combining a number of strategies. We extended the use of classical breeding with haploid derivatives of the industrial strains to breeding with diploid strains homozygous for the mating locus. This doubles the genetic reservoir for meiotic recombination. In addition, we evaluated large arrays of segregants, using multi-step selection procedures, first aimed at eliminating the truly inferior strains with simple high-throughput tests and gradually moving the selection to conditions as close as possible to the real industrial conditions. In addition, we submitted the candidate strains to very stringent independent evaluation tests, such as the ability to accumulate a very high titer of ethanol in semi-anaerobic very-high gravity fermentations. This is a stringent quality criterion for a superior bioethanol production strain.

We have used meiotic recombination to combine the superior elements from the genomes of the D-xylose utilizing strain GS1.11-26 and the highly inhibitor tolerant strain JT21653. The latter strain was identified through screening of a collection of various yeast strains from a variety of sources using undetoxified acid pretreated spruce hydrolysate. We chose this material since it has been reported to be among the most inhibitory hydrolysates, containing multiple inhibitors, including the furan derivatives furfural and HMF, aliphatic acids, such as acetic acid, formic acid and levulinic acid, and various phenolic compounds, such as vanillic acid, vanillin, synringaldehyde, syringic acid, and 4-hydroxybenzoic acid [24,29,44]. A strain tolerant to this medium would most likely also be tolerant to inhibitors in other lignocellulosic hydrolysates that are pretreated in a less severe way. Besides, acid based thermochemical pretreatment is considered to be one of the most cost-competitive methods of pretreatment for bioconversion of lignocellulosic feedstocks in liquid biofuel production [10,45]. Hence, such strains would be expected to have broad application potential.

Hybridization of industrial strains is among the most effective and simple techniques used for improving and combining various industrially relevant traits [46,47]. In a classical breeding strategy, haploid strains of opposite mating type are crossed to produce new diploid progeny. However, this method cannot be directly applied to industrial strains since most industrial strains are diploid, polyploid or aneuploid. Hence, the identification of a haploid meiotic segregant with in principle the same superior profile of traits as the parent industrial strain is required and this becomes exceedingly difficult when it concerns traits that are only important at industrial scale. Hence, the haploid progeny used for breeding will never display exactly the same repertoire of positive traits as the original diploid strain. This makes breeding of industrial yeast strains into a huge challenge. In our work, we have used for mating the original diploid *MATα/α* strain, GS1.11-26, rather than a haploid derivative, so as to maintain the entire genetic basis underlying the superior D-xylose fermentation capacity of that strain. Constructing hybrid strains by mating two mating competent diploid parents has been described previously [48]. However, in that report, no further meiotic recombination step had been applied to the tetraploid hybrid strains to isolate diploid segregants, which might be advantageous for two reasons. First, the most stable genome size in *S. cerevisiae* appears to be the diploid state. This is suggested by the frequent spontaneous evolution of ploidy from tetraploid to diploid in both optimal and stressful environments [49]. This implies that a tetraploid industrial strain will likely show lower stability and might easily lose important traits when losing chromosomes in the shift to a lower ploidy during its industrial usage. Second, meiotic recombination can generate great diversity in a cell population and sporulation of a tetraploid can therefore generate strains with superior performance compared to the tetraploid parent strain [50]. This was also observed in our work, in which diploid segregants were obtained with a much higher D-xylose utilization rate than the tetraploid parent. This is probably due to the involvement of recessive alleles in sustaining efficient D-xylose utilization.

We have used two approaches to improve the GS1.11-26 strain. The backcrossing with an Ethanol Red segregant was mainly aimed at eliminating the negative traits that had been introduced in the background of the strain during its development for high xylose fermentation capacity and inhibitor tolerance, since we maintained the original Ethanol Red background in the breeding process. This approach has been previously described as beneficial for weeding out deleterious mutations in mutant strains [51,52]. On the other hand, breeding with the Fseg25 strain introduced a new, albeit also industrial, genetic background and was mainly aimed at further enhancing inhibitor tolerance, with the risk of losing traits important for a bioethanol production strain. Both approaches were successful in generating strongly improved strains. The GSE16 strain had not only lost the low glucose utilization and aerobic growth rate of its parent, GS1.11-26, but also displayed inhibitor tolerance to a level at least as high as the original unevolved parent strain, HDY.GUF5. The GSF335 and GSF767 strains also largely lost the negative properties of their GS1.11-26 parent, but in addition gained at least part of the very high inhibitor tolerance of the Fseg25 parent strain.

The three hybrid strains were evaluated for various industrially relevant traits, including tolerance to different inhibitors, fermentation performance in a D-xylose/glucose mixture and in very-high gravity fermentation. All three new hybrid strains showed much better general performance in various stress conditions compared to GS1.11-26. There was no single strain that stood out in all conditions. The relative performance varied with the test. In a semi-anaerobic batch fermentation of spruce hydrolysate, the three strains showed a much shorter lag phase compared to GS1.11-26. On the other hand, the very high D-xylose fermentation capacity of GS1.11-26 was not fully maintained in the new hybrid strains. Even though the three hybrid strains were able to consume all the 37 g/L D-xylose and 36 g/L glucose in 32 h, they retained only 35% to 60% of the maximum D-xylose utilization rate of GS1.11-26. The higher inhibitor tolerance of the three new hybrid strains can explain why they displayed D-xylose utilization rates in spruce hydrolysate comparable to that of the strain GS1.11-26. The rate of glucose utilization was also significantly higher than that of GS1.11-26, which is likely also due to their higher tolerance to the inhibitors in spruce hydrolysate. Tolerance to multiple stress factors has previously been shown to correlate with high ethanol yield and a high ethanol production rate in *S. cerevisiae*[28]. Moreover, D-xylose fermentation is more sensitive to stress factors, especially to acetic acid, compared to glucose fermentation [26]. The severe reduction of tolerance to acetic acid (and possibly to other inhibitors) of GS1.11-26 [27] can explain its lower D-xylose utilization rate in spruce hydrolysate compared to synthetic medium. On the other hand, the rate of D-xylose utilization by the three new hybrid stains was not as severely reduced as for GS1.11-26 in the spruce hydrolysate compared to complex medium, which can be explained by their higher inhibitor tolerance.

Other diploid hybrid segregants with a higher D-xylose fermentation capacity than the three selected strains have been isolated, but they displayed very slow growth rates in glucose and also reduced inhibitor tolerance. Hence, they were excluded because of these negative properties. The difficulty in maintaining the superior D-xylose fermentation rate of the parent strain, GS1.11-26, might suggest that one or more of the negative background mutations in the strain GS1.11-26 might either be causally or structurally linked to the high D-xylose fermentation rate. If these traits are causally linked, the beneficial genes or loci important for efficient D-xylose fermentation might be linked with the reduced growth rate or with the higher inhibitor tolerance. This would mean that it will not be possible or be very difficult to combine high general robustness and high D-xylose utilization capacity in this strain background. However, if the negative mutations are only structurally linked to the positive genetic modifications, i.e. residing close to each other in the genome, they could be removed without affecting the superior D-xylose fermentation performance. Further research, therefore, should focus on the identification of the genetic basis of the efficient D-xylose fermentation as well as the high inhibitor tolerance, so that they can be engineered by reverse metabolic engineering.

Bioethanol production is commonly performed in very-high gravity fermentations using highly concentrated substrates so that a maximal final ethanol titer can be reached [53]. These substrates include first-generation feedstocks, such as sugar cane-molasses, starch or grains, and in the future, second-generation feedstocks, in particular lignocellulose waste steams and bioenergy crops. A high final ethanol titer has multiple advantages. It reduces the ethanol distillation costs but also lowers the liquid volumes in the plant causing large savings in heating, cooling, pumping and transport costs. Very-high gravity fermentation causes high stress, in particular osmostress in the beginning of the fermentation and ethanol stress at the end of the fermentation. This results in a longer fermentation time and lower ethanol yield as a result of higher residual sugar [54]. Hence, the performance of a new yeast strain in very-high gravity fermentation is a crucial quality criterion for its use in real industrial practice. This was especially important since the parent strain, GS1.11-26, was severely compromised in maximal ethanol accumulation capacity in very-high gravity fermentations [27]. Hence, we evaluated the three new hybrid strains also in very-high gravity fermentation and found that they were not only much better than GS1.11-26, but showed even improved performance in some cases compared to the original HDY.GUF5 strain. The hybrid strain, GSF335, exhibited significantly increased performance compared to its tetraploid parent as well as to both its diploid progenitor strains GS1.11-26 and Fseg25. This clearly shows that meiotic recombination with diploid strains is able to further improve important performance traits that are considered to be already very high in the very best currently used bioethanol production strains.

## Conclusions

We have successfully developed three robust industrial yeast strains that combine efficient D-xylose utilization with high inhibitor tolerance for use in bioethanol production with lignocellulose hydrolysates. Two of the strains (GSF3 35 and GSF767) have been derived through meiotic recombination of the efficient D-xylose utilizing strain GS1.11-26 (derived from Ethanol Red) with the most inhibitor tolerant strain obtained from a screening of more than 580 yeast strains. Strain GSE16 has a purely Ethanol Red background, developed by backcrossing GS1.11-26 with a haploid derivative of Ethanol Red. All three strains showed superior performance with respect to aerobic growth rate, glucose consumption rate and inhibitor tolerance compared to GS1.11-26. The D-xylose utilization rate of the three strains in complex medium was reduced compared to GS1.11-26, but in inhibitor-rich acid-pretreated spruce hydrolysate it was comparable. Due to the high robustness, the proven record in industrial application of the background strain Ethanol Red, and the efficient D-xylose utilization capacity, the three strains have strong potential for direct application in industrial bioethanol production. This study demonstrates that strains with an optimal profile of industrially important traits can be obtained through meiotic recombination directly with diploid strains and by screening large numbers of strains or segregants in a multi-step selection process, using simple high-throughput screens to eliminate poor performers to more elaborate evaluation conditions, mimicking closely the industrial conditions to select the very best performers.

## Methods

### Yeast strains and media

Yeast strains used in this study are listed in Table 1. Yeast cells were propagated in yeast extract peptone (YP) medium (10 g/L yeast extract, 20 g/L bacteriological peptone) supplemented with either 20 g/L D-xylose (YPX) or 20 g/L glucose (YPD). For preparation of solid plates, 15 g/L Bacto agar was added after adjusting the pH to 6.5 with 4 M KOH. Inocula for fermentation and growth tests were prepared by growing strains in YPD medium to stationary phase, harvesting by centrifugation at 2100 g for 5 min at 4°C, and washing with ice-cold sterile Milli-Q water.

**Table 1:** Yeast strains used in this study, including the 14 strains selected for highest inhibitor tolerance in spruce hydrolysate

**Name**	**Origin**	**Source/reference**
**JT21585**	Baker’s yeast	Puratos, Belgium
**JT21586**	Baker’s yeast	Puratos, Belgium
**JT21587**	Baker’s yeast	Puratos, Belgium
**JT21621**	Baker’s yeast	Anchor Yeast, South Africa
**JT21651**	Baker’s yeast	Algist Bruggeman, Belgium
**JT21652**	Baker’s yeast	Algist Bruggeman, Belgium
**JT21653**	Baker’s yeast	AB Mauri, Australia
**JT21698**	Distiller’s yeast	Lallemand, Canada
**JT22194**	Beer production yeast	Kasteel triple beer, Belgium
**JT22231**	Wine yeast	LALVIN (Lallemand, Canada)
**JT22272**	Natural isolate from apple vinegar	Natural isolate, Slovenia
**JT22280**	Natural isolate from apple vinegar	Natural isolate, Slovenia
**JT22728**	Baker’s yeast	PYCC-Portuguese Yeast Culture Collection, Portugal
**FY290**	Natural isolate from fruit	BCCM/MUCL (Mycothèque de l'Université Catholique de Louvain; Belgium)
**Ethanol Red**	Bioethanol production yeast	Fermentis, a division of S. I. Lesaffre, France
**HDY.GUF5**	Ethanol Red background, carrying D-xylose and L-arabinose metabolism gene cassette	[27]
**GS1.11-26**	HDY.GUF5 background, evolved for D-xylose fermentation	[27]
**Fseg25**	Segregant of JT21653	This study
**ER17**	Segregant of Ethanol Red	This study
**GSF335**	Hybrid between GS1.11-26 and Fseg25	This study
**GSF767**	Hybrid between GS1.11-26 and Fseg25	This study
**GSE16**	Hybrid between GS1.11-26 and ER17	This study

### Spruce hydrolysate

The spruce hydrolysate used in this study has been provided by SEKAB E-Technology AB (Örnsköldsvik, Sweden). It was prepared from spruce wood chips by pretreatment with SO_2_ impregnated steam explosion. The composition of the hydrolysate has been reported previously (20).

### Screening of strains for inhibitor tolerance in spruce hydrolysate medium

Initial screening of the yeast strain collection for inhibitor tolerance was performed on agar plates containing increasing concentrations of the whole slurry of spruce hydrolysate (40% to 70%) supplemented with yeast extract (10 g/L) and bacteriological peptone (20 g/L). The pH was adjusted to 5.5 using 4 M KOH. Further screening of the selected strains was performed under semi-anaerobic fermentation conditions using whole slurry of the pretreated spruce material diluted to 30% in YP medium and supplemented with glucose to a final concentration of 200 g/L, taking into account the initial glucose concentration already present in the hydrolysate. For screening of segregants from the most tolerant strain JT21653, the concentration of spruce hydrolysate was increased to 77% and, was supplemented with YP and glucose 70 g/L glucose. The fermentations were started at a cell density of 1.3 g/L, in 60 mL volume at 30°C with continuous stirring at 200 rpm. The apparent rate of fermentation was then followed by measuring the weight loss due to CO_2_ release.

### Screening segregants for growth and fermentation in D-xylose medium

Prescreening of segregants for the ability to grow in D-xylose medium was performed in a 24 well plate containing 1 mL of YP medium supplemented with 20 g/L D-xylose. Cells were inoculated at an initial OD_600_ of 1.0 from a preculture grown overnight in YPD medium. The OD_600_ was measured after 20 h cultivation at 30°C in an orbital shaker.

To select strains that were able to ferment D-xylose efficiently, small tube semi-anaerobic batch fermentations were performed at 35°C in 50 mL YP medium containing 40 g/L D-xylose as a carbon source. For this purpose, cylindrical tubes were used with a volume of 150 mL and fitted with a rubber stopper containing cotton-plugged glass tubing. The initial inoculum density was 1.3 g DW/L. The cultures were continuously stirred with a magnetic rod at 120 rpm. The rate of fermentation was estimated by following the weight loss due to CO_2_ release.

### Evaluation of strains for fermentation performance in a D-xylose-glucose mixture

For evaluation of the fermentation performance in medium containing D-xylose and glucose, the batch fermentations were performed in complex medium in 300 mL shake flasks with a working volume of 200 mL at 35°C. The initial inoculum density was 1.3 g DW/L. Flasks were closed with fermentation locks containing glycerol. Nitrogen gas was sparged after cell inoculation until the oxygen concentration reached about 2 ppm. Cultures were continuously stirred at 120 rpm using a magnetic stirrer. Samples were taken every few hours with needles for analysis.

### Evaluation of strains for fermentation performance in spruce medium

For evaluation of fermentation performance in spruce hydrolysate, the pretreated spruce material was used at a solid loading of 11% (corresponding to about 50% of the slurry) in 250 mL flasks with a working volume of 150 mL. Yeast Nitrogen base 1.7 g/L and ammonium sulfate (5 g/L) were added as supplement [21]. Since the pretreated spruce material was not enzymatically hydrolyzed, glucose and D-xylose were added to a final concentration of about 62 g/L and 18 g/L. The fermentation was started at an initial cell density of 4 g DW/L and incubated at 35°C with continuous stirring with a magnetic rod at 200 rpm. Samples were taken every few hours for analysis through plastic tubing fitted to the bottom side of the flasks, without introducing air.

### Very-high gravity fermentation

Very-high gravity fermentation was performed as previously described [55], in 150 mL volume cylindrical tubes containing 100 mL YP medium supplemented with 330 g/L glucose at 30°C. The prepared inoculum was resuspended in about 20–30 mL aliquot of the medium that was to be used for the fermentation, and inoculated into 100 mL final volume at an initial cell density of 1.3 g DW/L. Agitation was done with a magnetic rod at 120 rpm either continuously or for the first 4 h (in case of static fermentations). The fermentation rate was followed from the weight loss due to CO_2_ release. Samples were taken at the end for analysis.

### Determination of ploidy by flow cytometry

Flow cytometry analysis of DNA content was performed according to the method reported previously [56]. Briefly, exponentially growing cells were washed with ice-cold sterile water and fixed with 70% ethanol. Cells were treated with RNase (1 mg/mL) and the DNA was stained with propidium iodide (0.046 M) in 50 mM Tris, pH 7.7 and 15 mM MgCl_2_, at 4°C for about 48 h. The fluorescence intensity was measured using a FACScan instrument (Becton Dickinson).

### Meiotic recombination

Strains of opposite mating type were crossed by mixing small amounts of cells from each strain on a YPD plate. After 24 h incubation at 30°C, the cells were mixed again and re-incubated for another 24 h to increase the mating efficiency. The mixture was subsequently spread for single colonies on YPD plates. A few colonies were analyzed by PCR and flow cytometry to identify diploids. The selected *MAT****a****/α* diploids were sporulated in 1% potassium acetate for 5 to 7 days, at 23°C. Spores were isolated by tetrad dissection using an MSM micromanipulator (Singer instruments, Somerset, UK).

### Determination of mating type

Mating type was determined by PCR and pheromone assay. PCR was performed using a primer annealing to the MAT locus and a *MAT****a*** or *MATα* specific primer [57]. The mating type was further validated by a pheromone assay. For that purpose, small amounts of cells from two tester strains of *S. cerevisiae*, *MATa bar1-Δ* and *MATα sst2-Δ* were inoculated in 1% agar at 50°C in separate tubes. The mixture was immediately poured on top of a YPD plate. After the top agar solidified, about 10 μl of cell suspension from the strains to be tested, was spotted onto each tester plate and incubated for 24 h at 30°C. *MATα* cells showed a zone of growth inhibition on plates of the *bar1-Δ* strain while *MAT****a*** cells showed a zone of growth inhibition on plates of the *sst2-Δ* strain. Diploid cells did not produce a zone of inhibition.

### Bioscreen growth rate assay

To perform the growth rate assay, strains were first pregrown to early stationary phase in YPD medium. After washing the pellets in cold sterile water, the cells were inoculated into 200 μl synthetic medium containing 20 g/L glucose at an initial OD_600_ value of 0.1. The titer plates were incubated at 35°C with continuous shaking in a Bioscreen C reader (Labsystems) in which the OD_600_ values were monitored every 30 min.

### Analysis of cell mass and metabolite concentrations

The cell dry weight (DW) for inoculation into the fermentation medium was estimated based on the Optical Density (OD_600nm_). The DW was first measured by filtering a 10 mL culture aliquot with a known OD_600_ value over a preweighed 0.2 mm Supor Membrane disc filter (PALL Corporation, USA), washing the filter with MilliQ water, and drying it in a microwave oven at about 150 watt for 15–20 min to constant weight. The correlation between dry weight and the OD_600_ value was determined for each strain tested.

Metabolites and substrates in samples from fermentation experiments were analyzed by Waters Isocratic Breeze HPLC system (Waters, Milford, MA, USA) using ion-exchange column WAT010290 and a refractive index detection system (Waters 2414 RI detector). Column temperature was maintained at 75°C and 5 mM H_2_SO_4_ was used as eluent at a flow rate of 1 mL/min. For analysis of hydrolysate medium, samples were first centrifuged in 15 mL falcon tubes at 4000 g for 10 min. The supernatant was further centrifuged in 2 mL microcentrifuge tubes at 20,000 g for 5 min and then filtered using 0.2 μm filters. The filtrate was used for HPLC analysis. Ethanol from the very-high gravity fermentations was measured by near infrared spectroscopy (Alcolyzer, Anton Paar). Rates and yields were calculated as previously described [58].

## Abbreviations

DW: Dry weight; OD: Optical density; YPD: Yeast extract peptone dextrose; YPX: Yeast extract peptone D-xylose; HPLC: High performance liquid chromatography; PCR: Polymerase chain reaction.

## Competing interests

The authors declare that they have no competing interests.

## Authors’ contributions

MMD, FD, MRFM and JMT designed the experiments. MMD, YL and TB performed the experiments and MMD, FD, YL, TB, MRFM and JMT analyzed the results. MMD drafted the manuscript. FD participated in the drafting of the manuscript. MRFM and FD commented on the manuscript and provided academic supervision. JMT led the study and revised the manuscript. All authors have read and approved the final manuscript.
